# Nrf2, the Master Regulator of Anti-Oxidative Responses

**DOI:** 10.3390/ijms18122772

**Published:** 2017-12-20

**Authors:** Sandra Vomund, Anne Schäfer, Michael J. Parnham, Bernhard Brüne, Andreas von Knethen

**Affiliations:** 1 Fraunhofer Institute for Molecular Biology and Applied Ecology IME, Project Group Translational Medicine & Pharmacology TMP, Theodor-Stern-Kai 7, 60590 Frankfurt, Germany; sandra.vomund@ime.fraunhofer.de (S.V.); Michael.Parnham@ime.fraunhofer.de (M.J.P.); b.bruene@biochem.uni-frankfurt.de (B.B.); 2 Institute of Biochemistry I-Pathobiochemistry, Faculty of Medicine, Goethe-University Frankfurt, Theodor-Stern-Kai 7, 60590 Frankfurt, Germany; schaefer@biochem.uni-frankfurt.de

**Keywords:** Nrf2, electrophiles, reactive oxygen species, transcription factor, antioxidants

## Abstract

Tight regulation of inflammation is very important to guarantee a balanced immune response without developing chronic inflammation. One of the major mediators of the resolution of inflammation is the transcription factor: the nuclear factor erythroid 2-like 2 (Nrf2). Stabilized following oxidative stress, Nrf2 induces the expression of antioxidants as well as cytoprotective genes, which provoke an anti-inflammatory expression profile, and is crucial for the initiation of healing. In view of this fundamental modulatory role, it is clear that both hyper- or hypoactivation of Nrf2 contribute to the onset of chronic diseases. Understanding the tight regulation of Nrf2 expression/activation and its interaction with signaling pathways, known to affect inflammatory processes, will facilitate development of therapeutic approaches to prevent Nrf2 dysregulation and ameliorate chronic inflammatory diseases. We discuss in this review the principle mechanisms of Nrf2 regulation with a focus on inflammation and autophagy, extending the role of dysregulated Nrf2 to chronic diseases and tumor development.

## 1. Structure and Regulation of Nrf2

**Figure 1:**
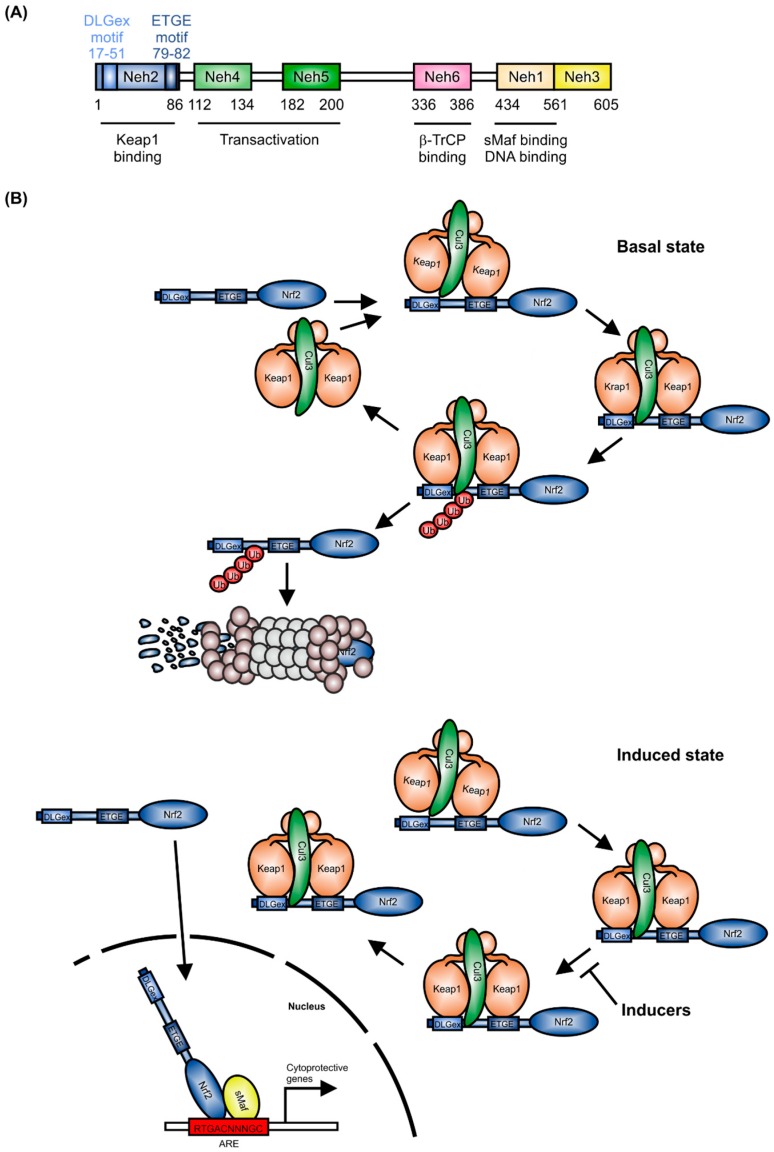

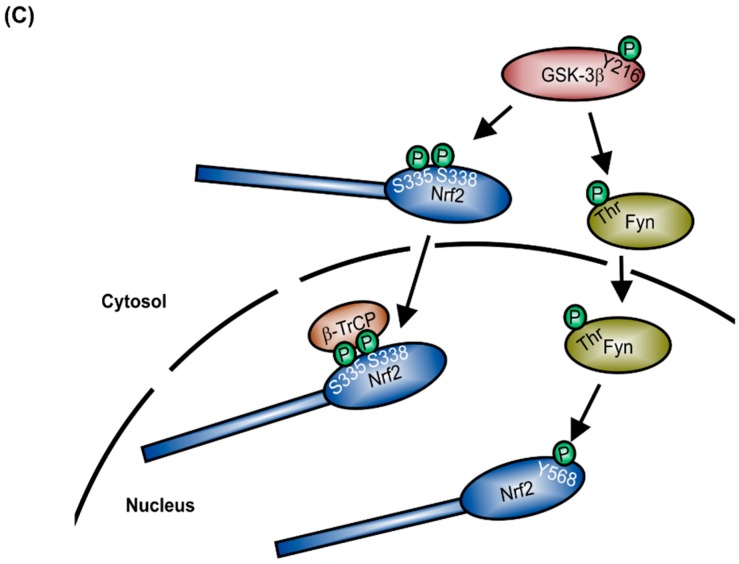
Regulation of Nrf2 expression. (**A**) Domain structure of Nrf2; (**B**) Keap1-dependent degradation of Nrf2 (mod. from [8]), T-bar = inhibition of Nrf2 degradation, consequently blocking Keap1 release; (**C**) Keap1-independent mechanism of Nrf2 degradation.

The CNC (cap-n-collar) subfamily of the basic region-leucine zipper-type transcription factor Nrf2 is encoded by the nuclear factor, erythroid-derived 2-like 2 (*NFE2L2*) gene [1]. As shown in Figure 1A, Nrf2 contains six Neh (Nrf2-ECH homology) domains. The Neh1 domain includes a basic region-leucine zipper (bZIP) structure, which is important for Nrf2 dimerization with small Maf proteins (sMafs) [2] and binding to DNA. Neh2 and Neh6 are degrons, which allow Nrf2 targeting to proteasomal degradation by Keap1 (Kelch-like ECH-associated protein1) and β-TrCP (β-transducin repeats-containing proteins) [3]. The Neh4 and Neh5 domains are responsible for Nrf2-dependent transactivation. 

As a master regulator of antioxidative responses, the expression of Nrf2 is tightly regulated. Under healthy conditions, Nrf2 mRNA is constitutively expressed [4] and Nrf2 expression is controlled at the protein level [5]. Following translation, Nrf2 is targeted by a Keap1 homodimer in the cytosol, which facilitates binding of the Cullin-3 (Cul3)/RING box protein1 (Rbx1) E3 ubiquitin ligase complex, consequently causing proteasomal degradation of Nrf2 [6] (Figure 1B). Binding of the Keap1 homodimer is mediated by the two Nrf2 sequences, ETGE and DLGex, located in the Neh2 domain [7]. In single live cells, it has been shown that in the control situation, the interaction of Nrf2 and Keap1 follows a cycle, whereby Nrf2 interacts with a single molecule of Keap1 (open confirmation, Figure 1B, basal state), followed by a closed conformation, in which Nrf2 binds to both members of the Keap1 dimer [8,9]. This closed conformation is stabilized by inducers (Figure 1B, induced state), without releasing Nrf2. Consequently, free Keap1 is not regenerated. Thus, de-novo synthesized Nrf2 is stabilized, translocates to the nucleus and induces expression of cytoprotective target genes [10]. These are mainly responsible for detoxification, antioxidation, and metabolism [11]. Moreover, Nrf2 inhibits activation of pro-inflammatory gene expression [12]. To fulfill its role as a transcription factor, Nrf2 binds, as a heterodimer with one of the sMafs, to the antioxidant-response-element (ARE) or electrophile-response element (EpRE) [13,14]. This is located in the promoter/enhancer regions of many cytoprotective genes.

After gene induction, Nrf2 is phosphorylated within the nucleus by the SRC family tyrosine kinase member, Fyn (Figure 1C). Fyn translocates to the nucleus in response to its threonine phosphorylation by active glycogen synthase kinase 3β (GSK-3β) [15]. There, activated Fyn phosphorylates Nrf2 at Tyr568, which leads to nuclear export and cytosolic degradation of Nrf2 [16]. Alternatively, in the cytosol, GSK-3β can also directly phosphorylate Ser335 and Ser338 of Nrf2, located in the Neh6 domain. This phosphorylated Nrf2 translocates to the nucleus and is recognized directly by β-transducin repeat containing E3 ubiquitin protein ligase (β-TrCP) (Figure 1C), provoking nuclear ubiquitination and degradation of Nrf2 [17]. Additionally to Nrf2 degradation as a mechanism to prevent Nrf2-dependent target gene induction, the BTB and CNC homology 1 (Bach1) transcription factor competes with Nrf2 for its binding sites on DNA, consequently suppressing Nrf2-mediated gene induction [18].

## 2. Nrf2 and Autophagy

The Nrf2-Keap1 signaling pathway and its subsequent induction of cytoprotective protein expression is the main cellular defense mechanism against oxidative and electrophilic stress. Therefore, interactions of Nrf2 with other signaling components regulate the efficiency of the cellular stress response. One of these is macroautophagy (hereafter referred to as autophagy). In general, autophagy preserves cellular homeostasis and protects against oxidative and proteotoxic stress by clearance of damaged organelles, long-lived proteins, as well as misfolded and aggregated proteins. This intracellular bulk degradation is executed through engulfment by double-membrane vesicles, named autophagosomes, which fuse with lysosomes [19]. Autophagy can be non-selective, especially induced by starvation, but there is also a basal autophagy that selectively sorts proteins and unwanted organelles to maintain cellular homeostasis [20].

**Figure 2:**
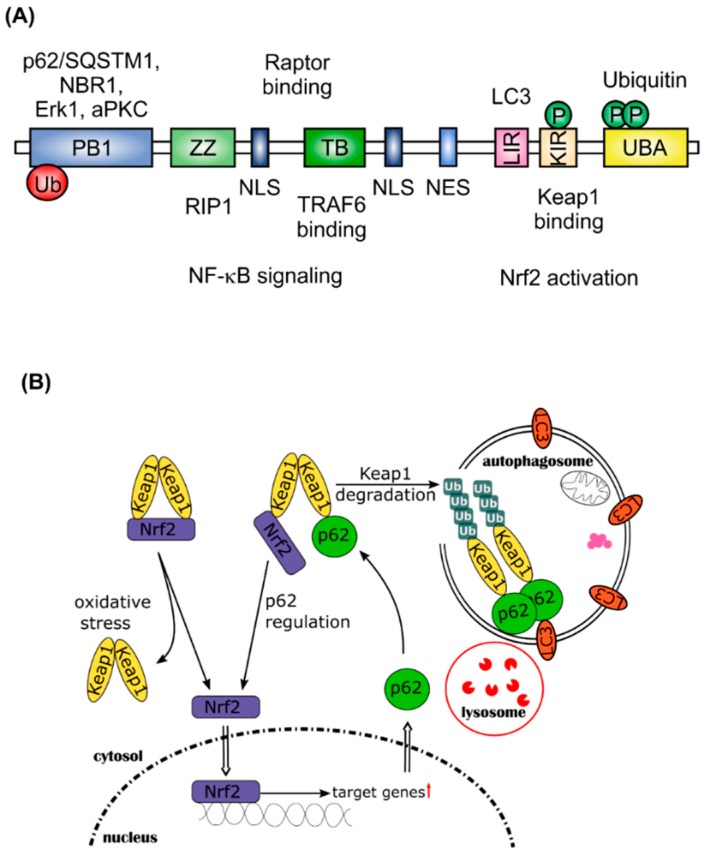
Positive feedback-loop of Nrf2 activation by *p62/SQSTM1*. (**A**) Domain structure of p62/SQSTM1; (**B**) p62/SQSTM1 is an important protein for selective autophagy, binds to Keap1 and other long-lived proteins and forms polyubiquitinated protein aggregates. Furthermore, it binds to the autophagy marker LC3 within the autophagosome, thereby leading the aggregated proteins into the autophagosome. After fusion with a lysosome, proteins and organelles, such as mitochondria, are degraded within the autophagosome. By binding to Keap1, p62/SQSTM1 stabilizes Nrf2 and enhances its translocation into the nucleus, where Nrf2 activates its target genes (↑ = upregulation of Nrf2 target genes). One of these genes is *p62/SQSTM1*.

Nrf2 activation interferes with selective autophagy. In this case, the Nrf2 inhibitor Keap1 affects p62/sequestosome 1 (SQSTM1)-dependent signaling [21]. The p62/SQSTM1 protein is a ubiquitously expressed cellular protein, also known as A170, found in inclusion bodies containing polyubiquitinated protein aggregates. Through binding to microtubule-associated protein 1 light chain 3 (LC3), a well characterized autophagy marker, p62/SQSTM1 leads these inclusion bodies into the autophagosome where the proteins are digested (Figure 2). Therefore, p62/SQSTM1 acts as an adaptor between polyubiquitinated proteins and autophagy. As shown in Figure 2A, the domain structure of p62/SQSTM1 facilitates the binding of various proteins, such as Raptor, extracellular regulated kinase 1 (Erk1), atypical protein kinase C (aPKC), TNF-receptor associated factor 6 (TRAF6), receptor-interacting serine/threonine-protein kinase 1 (RIP1), and Keap1. This multifunctionality points to an important role of p62/SQSTM1 in intracellular signaling [22]. Inclusion bodies have been found to accumulate in several human diseases, especially in neurodegenerative diseases (Huntington disease, Parkinson disease, Alzheimer disease, and amyotrophic lateral sclerosis), but also in liver disorders, such as alcoholic hepatitis, steatohepatitis, and cancer [23,24,25]. Komatsu and co-workers have shown that suppression of autophagy leads to the formation of ubiquitin-positive inclusion bodies in mouse models [26,27]. This group also found that NAD(P)H quinone dehydrogenase 1 (Nqo1) and glutathione *S*-transferase (Gst), as Nrf2-regulated proteins, accumulate together with p62/SQSTM1 in autophagy-deficient mice and that Keap1 directly interacts with p62/SQSTM1, to be degraded within the autophagosome. Binding of p62/SQSTM1 to Keap1 occurs at the Kelch domain of Keap1, which is also one of the binding sites for Nrf2. Therefore, accumulation of p62/SQSTM1, for example due to autophagy deficiency, sequesters Keap1 and results in the release and stabilization of Nrf2 [28]. Nrf2 then translocates into the nucleus and activates expression of its target genes. One of these is *p62*/*SQSTM1* itself, pointing to a positive feedback loop in the activation of Nrf2 and initiation of selective autophagy via *p62*/*SQSTM1* [29]. This feedback loop is problematic for cancer therapy. Chemotherapy induced autophagy is thought to protect cancer cells from apoptosis. Nrf2 activation promotes the resistance of cancer cells to stress induced by chemotherapeutic drugs [30,31]. Thus, autophagy deficiency and consequent accumulation of p62/SQSTM1 is suspected to be tumorigenic [32].

## 3. Nrf2 in Inflammation

Based on its function as the master regulator of antioxidant signaling, Nrf2 is also involved in the regulation of pro- vs. anti-inflammatory gene expression. In line with this role, in a murine acute inflammation setting, Nrf2 knockout significantly exacerbated the condition [33], favouring the development of autoimmunity [34]. In inflammation, macrophages are crucial mediators of the innate immune response and show remarkable phenotype plasticity [35]. Thus, following classical inflammatory activation in response to interferonγ-(IFNγ)- and/or lipopolysaccharide-(LPS)-treatment, a pro-inflammatory M1-like macrophage phenotype is generated [36]. In contrast, after alternative activation with IL-4, IL-13, or glucocorticoid treatment, an anti-inflammatory, wound-healing promoting, M2-like macrophage phenotype is observed [37].

Because generation of reactive oxygen species (ROS) is an important characteristic of pro-inflammatory responses in phagocytes [38,39], Nrf2 is mainly an anti-inflammatory mediator, confining inflammatory responses [40]. One explanation for the anti-inflammatory effect of Nrf2 is its ability to inhibit the expression of pro-inflammatory cytokines, like TNF-α and IL-6, as well as inducible nitric oxide synthase (iNOS). Nrf2-dependent induction of antioxidant target genes, such as heme-oxygenase-1 (*HO-1*), *Nqo1*, glutamate cysteine ligase catalytic (*Gclc*) and modifier (*Gclm*) subunits, blocks the activation of the classical pro-inflammatory transcription factor NF-κB, thereby inhibiting the transcription of pro-inflammatory mediators [41,42,43]. Moreover, recent data support an inhibitory effect of Nrf2 on pro-inflammatory gene expression by directly blocking transcription. Thus, Nrf2 binds to the promoter region of pro-inflammatory genes (*IL-6*, *IL-1β*) and inhibits RNA polymerase II recruitment [12].

Interestingly, Nrf2 gives rise to a novel macrophage phenotype, named Mox [44]. Following treatment with oxidized phospholipids, macrophages in a murine model differentiate to the Mox phenotype and play an important role in the development of atherosclerotic lesions. The generation of this phenotype was prevented in macrophages derived from Nrf2-deficient mice. In this context, the *Mox* gene-expression profile was compromised and the regulation of the redox status was impaired. These in vivo data are supported by mechanistic data obtained in vitro in RAW264.7 macrophages, in which Nrf2-dependent counteraction of LPS-mediated inflammatory responses in foam cell macrophages was observed [45].

Nrf2-dependent gene expression also alters responses of adaptive immune cells. In a cytotoxicity assay, using macrophages derived from Nrf2 knockout mice and wild type cytotoxic T cells (CD8^+^), cytotoxicity was significantly reduced. Mechanistically, it was shown that in Nrf2-deficient macrophages, expression of the Nrf2-target genes *Gclm* and cystine antiporter *xCT* are inhibited. These enzymes are important for synthesis of thiols, which are released from the macrophages into the medium. Because CD8^+^ T cells do not express these Nrf2-target gene enzymes, which are required for efficient CD8^+^ T cell activity, the T cells need to take up the thiols from the medium. Consequently, cytotoxicity of wild type CD8^+^ T cells towards antigens presented by Nrf2-deficient macrophages was significantly reduced [46].

Besides its gene regulatory function, Nrf-2 is important in NLR family pyrin domain containing 3 (NLRP3) inflammasome activation and concomitant IL-1β expression [47]. NLRP3 cleaves caspase-1, initiating the processing of pro-IL-1β to mature IL-1β, which is then released from the cells [48]. Nrf2 blocks NLRP3 activation by induction of Nqo1 expression, which inhibits reactive oxygen species (ROS)-dependent NLRP3 priming [49]. In line with this action, Nrf2 activation by dimethyl fumarate, epigallocatechin-3-gallate, citral, mangiferin, or biochanin A prevents NLRP3 activation, consequently reducing the pro-inflammatory outcome [50,51,52,53,54]. However, Zhao et al. showed that in Nrf2-deficient macrophages, maturation and secretion of caspase-1 and IL-1 is significantly reduced following stimulation with NLRP3 activators, compared to wild-type controls [55]. Moreover, in the apolipoprotein E knockout mouse model, Nrf2 deficiency inhibits cholesterol crystal-induced inflammasome activation. This is accompanied by an inhibition of pro-atherogenic cytokine expression, i.e., IL-1β [56]. From these data, it can be concluded that Nrf2 is an important regulator of processes leading to toxicity and inflammatory diseases.

## 4. Nrf2 and Mitochondrial Function

Recent studies identified a new function of Nrf2 in regulating mitochondrial function. Nrf2 is involved in regulation of mitochondrial membrane potential and the availability of substrates for respiration and ATP synthesis. Based on a proteomics study in the human breast epithelial cell line MCF10A, Nrf2-dependent upregulation of the mitochondrial electron transport chain component, NDUFA4, in response to pharmacological Nrf2 activation by sulforaphane was observed, whereas Keap1 knockdown, leading to Nrf2 upregulation, downregulated expression of cytochrome c oxidase subunits COX2 and COX4I1 [57]. In a proteome derived from primary murine liver, it was shown that Nrf2 activation modulates expression of ATP synthase subunit α [58]. Following oxidative stress, Nrf2-mediated expression of the uncoupling protein 3 increased proton conductance of the inner mitochondrial membrane to reduce superoxide formation [59]. Moreover, in the absence of Nrf2, NADPH levels are reduced. This is mainly mediated by decreased expression of malic enzyme 1, isocitrate dehydrogenase 1, glucose-6-phosphate dehydrogenase, and 6-phosphogluconate-dehydrogenase. These genes are positively regulated in Nrf2 wild type cells. Less NADPH provokes reduced conversion of oxidized to reduced glutathione, which finally results in a shift to an oxidized state of the cells, consequently leading to inflammation and cell death [60,61].

Nrf2 is also important in mitochondrial biogenesis. Using the ratio of nuclear to mitochondrial DNA as readout system, it has been observed that in Nrf2-deficient livers, the amount of mitochondrial DNA is significantly lower than that in wild-type cells [62]. Transcriptional coactivators, such as peroxisome proliferator-activated receptor γ coactivator 1α, which are important in initiating protein synthesis during mitochondrial biogenesis, are also affected by Nrf2 [63,64]. Taking into consideration that nucleotide supply during mitochondrial biogenesis is also mandatory, it is of note that Nrf2 induces purine synthesis by upregulating the pentose phosphate pathway [65].

Finally, Nrf2 seems to be involved in controlling mitochondrial integrity during inflammation and oxidative stress. It has been shown that Nrf2 activation by sulforaphane protects mitochondria, isolated from the brain of rats, from opening of the mitochondrial permeability transition pore in response to tert-butylhydroperoxide treatment [66,67]. Mechanistically, this resistance is accompanied by a sulforaphane-dependent increase in mitochondrial glutathione, glutathione peroxidase 1, malic enzyme 3, and thioredoxin 2 [67]. During oxidative stress, Nrf2 might also contribute to the regulation of mitophagy, a process by which damaged mitochondria are removed, via regulation of autophagosomal degradation [68]. This effect was observed in a murine sepsis model, in which an increase in MAP1 light chain 3-II and the adaptor protein p62/SQSTM1 was suppressed, compared to wild type mice, in animals without functional expression of Nrf2 [69]. From these data, it can be concluded that Nrf2 has an important function in mitochondrial regulation.

## 5. Nrf2 in Toxicity and Disease

### 5.1. Nrf2 in Acetaminophen-Induced Liver Injury

The cytoprotective capacity of Nrf2 plays an important role in protection of the liver. The liver, as the main organ for biotransformation and subsequent detoxification of xenobiotics, has a high metabolic rate and is, therefore, prone to chemical and oxidative stress [70]. As a transcription factor inducing the expression of enzymes involved in biotransformation and detoxification, Nrf2 plays a key role in the elimination of drugs, toxic metabolites and other harmful xenobiotics [71]. Acetaminophen (APAP) is an example of a drug with a toxic metabolite that can lead to liver failure [72,73,74]. APAP is widely used as an analgesic to treat pain and fever. If taken in recommended doses, it is considered to be safe and effective, but due to its relatively narrow therapeutic index, overdoses of a single dose or excessive daily doses as a result of self-medication lead to hepatocyte injury and acute liver failure [75]. APAP intoxication accounts for the most common form of liver failure in the United States.

**Figure 3:**
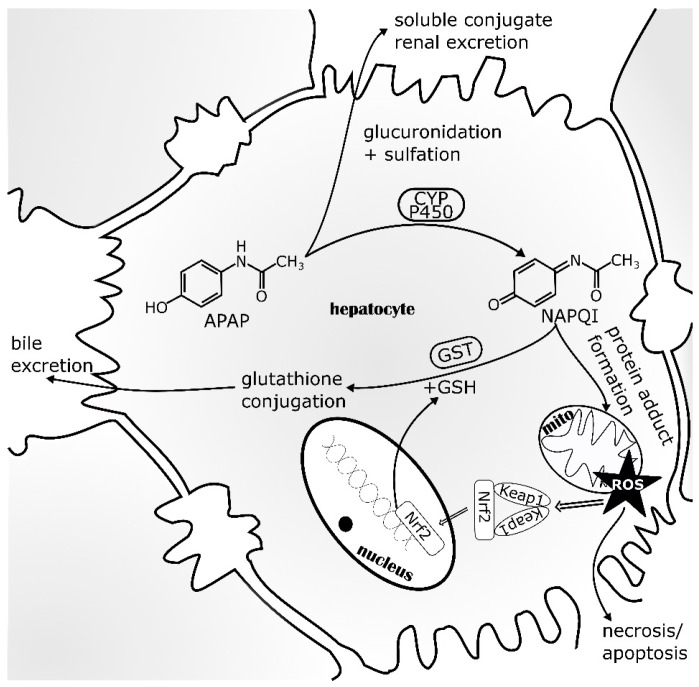
Acetaminophen (APAP) metabolism within the liver. Entering hepatocytes, APAP is metabolized >80% by glucuronyltransferases and sulfotransferases to soluble conjugates, which are excreted into the urine. CYP P450 monooxygenases can metabolize APAP to the electrophilic reactive metabolite NAPQI that is detoxified by glutathione *S*-transferase (Gst) using glutathione (GSH). If GSH stocks are exhausted, NAPQI oxidizes liver proteins, especially mitochondrial proteins by covalent binding. This induces ROS generation and consequently oxidative stress, which can lead to hepatocyte necrosis and apoptosis. To counteract cell death, cytoprotective signaling via Nrf2 activation and stabilization is induced by oxidative stress. Thereby, Keap1 releases Nrf2 which translocates into the nucleus, induces cytoprotective gene expression and replenishes the GSH stores. APAP—acetaminophen; CYP—cytochrome P450; GSH—glutathione; Gst—glutathione S-transferase; mito—mitochondria; Keap1—Kelch-like ECH associated protein; NAPQI—*N*-acetyl-p-benzoquinone imine; Nrf2—NF-E2 p45-related factor 2; ROS—reactive oxygen species (adapted from [85]).

At recommended doses, APAP is 80% metabolized to APAP-glucuronide and APAP-sulfate. These metabolites are then excreted into the urine. If this direct conjugation by glucuronyltransferases and sulfotransferases is saturated, as a result of glutathione (GSH) depletion, APAP is metabolized by cytochrome (CYP) P450 monooxygenases, predominantly CYP2E1. This leads to the formation of *N*-acetyl-p-benzoquinone imine (NAPQI), an electrophilic reactive metabolite. NAPQI is detoxified by conjugation with GSH via phase II metabolism by glutathione *S*-transferase (GST) and then excreted as the NAPQI-GSH conjugate through the biliary tract. After GSH depletion, NAPQI as a strong oxidizer covalently binds to intracellular liver proteins, especially those in the mitochondria (Figure 3). This binding of NAPQI to mitochondrial proteins leads to ROS formation, causing oxidative stress to the cell and ultimately provoking necrotic cell death [76]. To date, clinical treatment of patients with APAP intoxication relies heavily on *N*-acetylcysteine (NAC). As a hepatocyte accessible precursor for GSH, NAC replenishes cellular GSH stores and detoxifies NAPQI. If treatment is started shortly after intoxication, ideally within 12 h, NAC prevents hepatic injury [77]. The ability of Nrf2 to induce GSH production and thus, protect cells from oxidative stress, is utilized for drug development. For example, oleanolic acid (OA), used to treat liver diseases for many years, has been shown to protect against APAP intoxication and other types of chemically-induced acute necrotic liver injury in animal studies [78,79]. OA was further developed to synthetic derivatives like 2-cyano-3,12-dioxo-oleana-1,9(11)-dien-28-oic acid (CDDO), CDDO-methyl (CDDO-Me) and CDDO-imidazole (CDDO-Im), which have been shown to be potent inducers of Nrf2-regulated genes [80,81]. CDDO-Me has been investigated in clinical trials for diverse indications, although a phase III study for chronic kidney disease was terminated due to increased risk of heart failure in stage 4 patients [82]. Other natural products, known for their liver protective potential, might also act via the Nrf2 signaling pathway and it will be very interesting to further investigate their potential as drugs to treat APAP intoxication [83,84].

### 5.2. Nrf2 in Chronic Obstructive Pulmonary Disease (COPD)

CDDO-Me is currently in use in a phase III study in patients with pulmonary hypertension (PH), showing the importance of Nrf2 activation also for lung diseases (LARIAT, NCT02036970) [86]. Lung epithelial cells are directly exposed to environmental irritants from air borne pollutants or tobacco smoke, which cause oxidative stress and inflammatory response and subsequently, can lead to alveolar cell apoptosis and emphysema, characteristics of chronic obstructive pulmonary disease (COPD). In patients with COPD, Nrf2 expression is reduced, consequently attenuating Nrf2-dependent expression of cytoprotective genes [87,88]. Likewise, Nrf2-deficient mice are more vulnerable to emphysema [89,90].

COPD is associated with persistent abnormal inflammation in the lung, accompanied by an accumulation of neutrophils, macrophages, dendritic, CD81, CD41, NK, and B cells [91]. Excessive oxidative stress, for instance, due to smoking, amplifies inflammation and induces death of structural cells in the lung by enhancing gene expression of pro-inflammatory mediators [92]. The Nrf2 antioxidant response attenuates this oxidative stress and exerts protection against oxidative stress-driven pathophysiology in patients with COPD. Surprisingly, pharmacological antioxidants such as NAC showed little or only modest effects. Therefore, Nrf2 activation seems to have additional effects apart from reduction of oxidative stress. Recently, it has been shown, that Nrf2 suppresses the inflammatory response of macrophages by blocking pro-inflammatory cytokine transcription [12]. This finding might be crucial in understanding the function of Nrf2 in relation to inflammation and indicates that the role of Nrf2 is not limited to control of oxidative stress and inflammation through ROS elimination. It may also be a negative regulator of the expression of pro-inflammatory cytokines, such as interleukin IL-6 and IL-1β [12]. Furthermore, Nrf2 regulates expression of the macrophage scavenger receptor with collagenous structure (MARCO), which facilitates binding and uptake of bacteria, oxidized low-density lipoproteins, and environmental particles [93]. MARCO is highly expressed in lung-resident macrophages, demonstrating the importance of Nrf2 activation in phagocytic bacterial clearance by alveolar macrophages [94,95].

**Figure 4:**
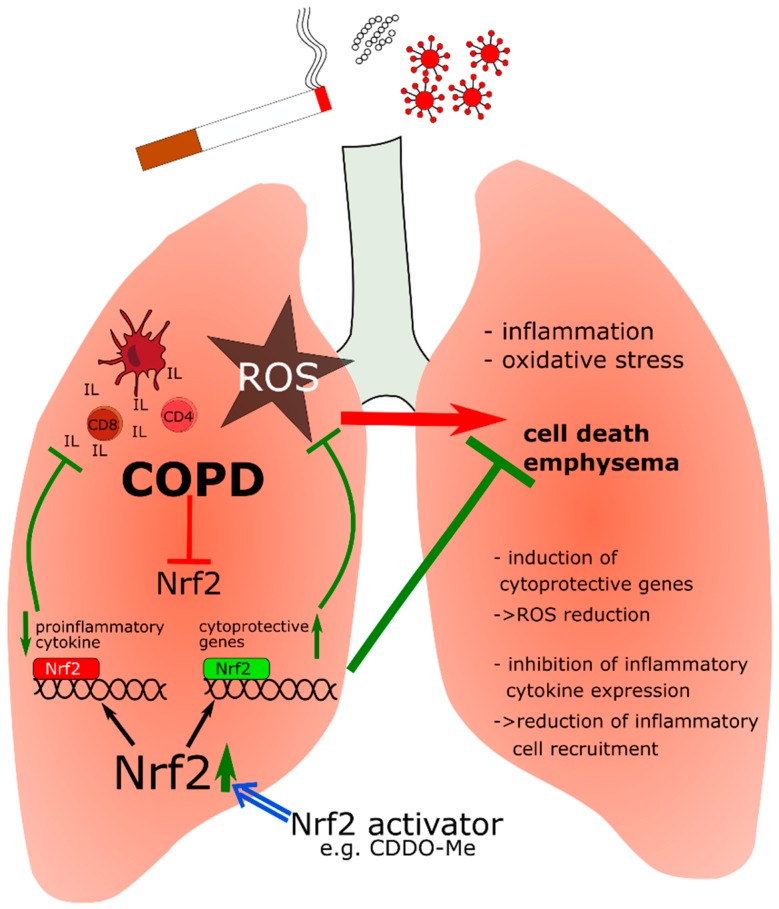
Influence of Nrf2 activation on COPD. Air pollutants, cigarette smoke and bacterial or viral infection cause oxidative stress and inflammation in the lung. Nrf2 activation induces cytoprotective gene expression to counteract the toxic effect of ROS. Moreover, Nrf2 inhibits transcription of proinflammatory cytokines, especially in macrophages, to reduce the recruitment of inflammatory cells into the lung. If Nrf2 is reduced, inflammation and ROS lead to cell death of lung epithelial cells and consequently mediate emphysema. CD4/CD8—effector/cytotoxic T-cells; IL—interleukins; —NF-E2 p45-related factor 2; ROS—reactive oxygen species, ↑/↓ = up-/downregulation of expression with a positive effect on disease progression, T-bars/T-bars = inhibition with a positive/negative effect on disease progression.

Taken together (Figure 4), Nrf2 has an important function in COPD. The fact that Nrf2 is diminished in the lungs of COPD patients points to the therapeutic potential of Nrf2 activators.

### 5.3. Cancer (Constitutive Nrf2 Activation)

Reactive oxygen species (ROS) are implicated in cancer and tumorigenesis in diverse ways. ROS can be involved in cancer initiation by causing DNA damage, which in turn, can lead to mutations and uncontrolled cell proliferation [96]. On the other hand, ROS are also involved in tumor growth and progression [97,98]. Considering the importance of ROS in tumorigenesis, it is hardly surprising that Nrf2, as the major regulator of redox homeostasis, plays a relevant role in the processes of cancer initiation and progression.

Mice deficient in Nrf2 are more vulnerable to environmental and intracellular stresses causing DNA damage and cancer. Treated with the carcinogen, benzo[a]pyrene, Nrf2 knockout (KO) mice exhibited a significantly higher gastric tumor burden compared to wild-type mice [99]. Similarly, the incidence and size of colorectal tumors, in a colitis-associated carcinogenesis model, were increased in Nrf2-deficient mice [100]. Furthermore, Aoki et al. [101] showed that the formation of DNA adducts upon exposure to diesel exhaust is increased in Nrf2 KO mice, making them more susceptible to mutations and carcinogenesis. The accelerated DNA-adduct formation is explainable by the low activity of phase II drug-metabolizing enzymes, as a result of the lack of transcription factor Nrf2. Clearly, activation of Nrf2 can ameliorate the onset of oncogenesis.

However, there is a dark side to Nrf2. Many types of cancer exhibit aberrant levels of Nrf2, as a result of the dysregulation of the Keap1-Nrf2 pathway. Correspondingly, many mutations within *Keap1* and *Nrf2* genes have been identified in several cancers, as elegantly reviewed by Taguchi, et al. [102]. All mutations described in *Nrf2* are located either within the DLGex or the ETGE motifs, preventing regular Keap1 binding and subsequent proteasomal degradation. Besides the obstruction of the Keap1-Nrf2 pathway by mutations, transcriptional alterations have also been observed. Hypermethylation of the *Keap1* promoter is observed in lung, prostate and kidney cancer [103,104,105]. The decreased *Keap1* transcription and thereby reduced expression, leads to elevated Nrf2 synthesis and activity.

The binding capacity of Keap1 to Nrf2 can also be decreased despite its unrestricted expression. Recently, Ge, et al. [106] showed that the oncogenic potential of the inhibitor of apoptosis-stimulating protein of p53 (iASPP), a known inhibitor of the tumor suppressor p53, is based on its ability to compete with Nrf2 for Keap1-binding. They demonstrated that the knockdown of iASPP leads to sensitization of renal cell carcinoma cells to the cytostatic agent 5-fluorouracil (5-FU). The effect of this increased 5-FU susceptibility is abolished by simultaneous knockdown of Nrf2.

Similarly to iASPP, p62/SQSTM1 can interfere with Keap1-Nrf2 binding. Impaired autophagy and the resulting accumulation of p62/SQSTM1 leads to an increase in Nrf2 activity. p62/SQSTM1 competes with Nrf2 for binding to Keap1, thereby leading to decreased degradation of Nrf2 and increased activity [28,107,108]. Inami, et al. [109] also reported that persistent Nrf2 activation, due to the accumulation of p62/SQSTM1, is involved in hepatoma development in Atg7-deficient mice.

Increased Nrf2 activity was also observed upon expression of the oncogenes *Kras*, *Braf*, and *Myc* in primary murine cells and human pancreatic cancer [110]. Kras-dependent upregulation of Nrf2 target genes, seen in non-small cell lung cancer (NSCLC) by Tao, et al. [111], further indicates an oncogene-dependent induction of Nrf2 activity.

This increased Nrf2 activity in tumors leads to an increase in target genes, which mainly account for the pro-tumorigenic properties of Nrf2. The ARE-regulated antioxidant target genes of Nrf2 allow cancer cells to maintain elevated ROS levels for pro-tumorigenic cell signaling and proliferation, without leading to ROS-mediated cell death [112]. In addition, Nrf2 was reported to upregulate Bcl-2 and Bcl-xL, preventing apoptosis and promoting drug resistance [113,114].

Nrf2-dependent chemoresistance is reflected by enhanced resistance of cancer cells to chemotherapeutics when Nrf2 is stably overexpressed [115]. In contrast, the inhibition of Nrf2 by either Keap1 overexpression, Nrf2-small interfering RNA or by an Nrf2 depleting drug leads to anti-cancer drug susceptibility of these cells [115,116]. Therefore, the role of Nrf2 in tumorigenesis depends on the phase (initiation or progression) and etiology of the disease.

**Figure 5:**
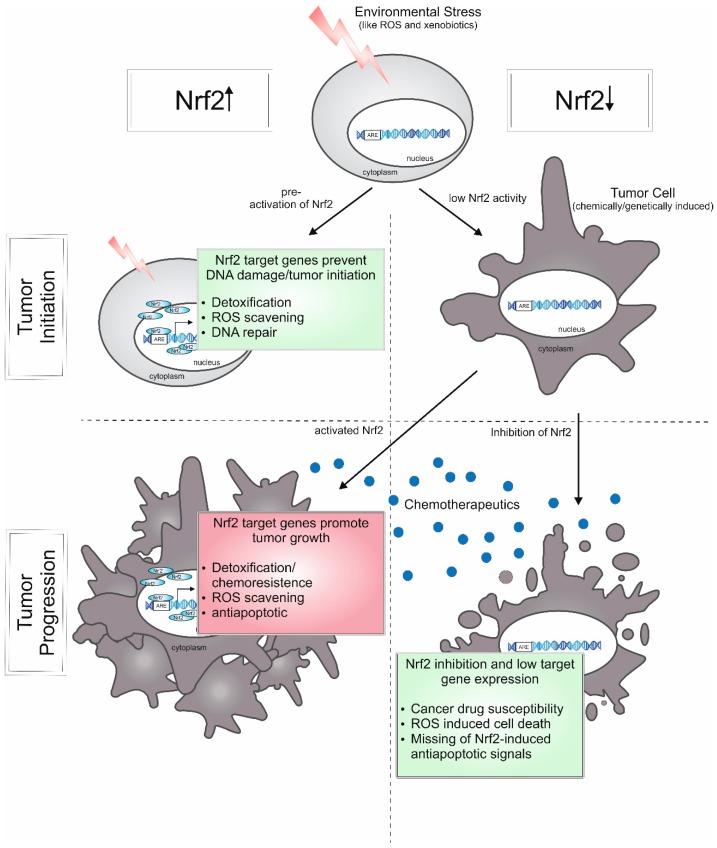
Roles of Nrf2 in tumorigenesis. Activated Nrf2 can prevent onset of cancer by protecting the cell from environmental stressors, like ROS and xenobiotics which can cause DNA damage. Low levels of Nrf2-target genes (reducing ROS levels and eliminating xenobiotics) can lead to tumorigenesis upon stress. In contrast, during tumor progression, Nrf2 activity can contribute to chemoresistance and the ability of tumor cells to circumvent apoptosis, whereas the inhibition of Nrf2 provokes cancer drug susceptibility and the loss of antiapoptotic signals.

These contrasting roles of Nrf2 are strikingly demonstrated by data from the group of Donna D. Zhang in a recent study [117] and summarized in Figure 5. Pre-treatment with the Nrf2 activator sulforaphane provokes smaller and fewer tumors in chemically-induced lung cancer, but has no effect on tumor burden in genetically-induced lung cancer. Nevertheless, Nrf2 inhibition is effective as anti-cancer intervention in both cancer models.

Accordingly, Nrf2 activation and antioxidants are able to reduce the risk of environmentally-induced cancer, but as cancer treatment, the inhibition of Nrf2 in combination with chemotherapeutics seems to be a promising approach. Therefore, there is a need to identify therapeutics and mechanisms to inhibit Nrf2 activity.

## 6. Clinical Potential of Pharmacological Modulation of Nrf2

Peng, et al. [118] identified in an ARE-luciferase assay, that among others, the antibiotics ethionamide and isoniazid impair Nrf2 activity. They demonstrated that disrupting the Nrf2–ARE pathway sensitizes human acute monocytic leukemia cells towards chemotherapeutics.

A positive effect was also obtained by combined treatment with brusatol and cisplatin of the lung cancer cell line A549, which exhibits high levels of Nrf2 due to a mutation in Keap1 [119]. The quassinoid, brusatol, was identified in an ARE-luciferase screen and was shown to transiently deplete Nrf2 in a concentration-dependent manner [119,120]. However, the inhibiting effect of brusatol is not Nrf2 specific, but rather due to inhibition of cap-dependent as well as cap-independent protein translation [121]. Therefore, therapeutic application of brusatol could be a problem as it also affects other short-lived proteins. In a search for specific Nrf2 inhibitors, Singh, et al. [122] identified a small molecule that selectively suppresses Nrf2 activity by binding to the Neh1 domain. In addition, they demonstrated that this molecule, in combination with platinum-based cancer drugs, increases the drug-induced cytotoxicity in Keap1-deficient NSCLC tumors compared to single agent treatment.

A more radical chemotherapeutic strategy to disturb tumor capacity by dealing with elevated ROS levels, was shown in head and neck cancer (HNC). By simultaneously targeting the glutathione-(GSH), thioredoxin-(Trx) and Nrf2-system, Roh et al. [123] achieved growth inhibition in resistant HNC.

Instead of targeting Nrf2 directly, therapeutic approaches are also exploiting the resulting phenotype of a dysregulated Keap1-Nrf2 pathway. Recently, it was reported that *Kras*-mutant lung adenocarcinoma, with a loss-of-function in the *Keap1* gene, shows enhanced dependency on glutaminolysis. Consequently, growth of *Keap1*-mutant lung tumors was markedly reduced upon inhibition of glutaminase [124].

Additionally, Nrf2 has been shown to modulate anabolic metabolism in cancer to allow tumor cell proliferation. This is achieved by redirecting glucose and glutamine into anabolic pathways [65]. In general, the constitutive supply of biosynthetic components, which guarantees the formation of proteins, lipids, and nucleotides, is a prerequisite for cell proliferation. Nrf2 induces the expression of enzymes of the pentose phosphate pathway [65,125], nucleotide biosynthesis [65], amino acid metabolism [57,126,127], and NADPH synthesis [128,129]. From these data, it is obvious that pharmacological activation of Nrf2 shapes the cell’s metabolic phenotype, leading to cancer cell growth and proliferation.

Taken together, targeting the Nrf2-redox-system in combination with chemotherapeutics could be a promising treatment for several cancers. Nevertheless, the success of these therapies depends on the identification of specific inhibitors. Moreover, it will be important to specifically target tumor cells to maintain the Nrf2-ARE pathway intact in healthy tissue, thereby protecting it from damage by environmental stressors, xenobiotics and ROS and preventing further malignant degeneration.

In addition to the use of CDDO-Me for APAP intoxication and the mechanistic basis for Nrf2 activation in COPD, there is an extensive literature on the potential for therapeutic modulation of Nrf2 in inflammatory and immunological disorders [130]. Many of the agents shown to activate Nrf2 are plant-derived antioxidant phenolic agents, such as flavonoids, which activate Nrf2 by interacting as Michael acceptors with thiol groups (e.g., on cysteine) expressed on the cell surface [131]. They thus act in a non-specific manner to regulate ROS production and, since they occur in foods, have been proposed as prophylactic dietary supplements for a variety of diseases. On the other hand, some synthetic agents, such as CDDO-Me, have been reported to have potential therapeutic relevance. Although effective in several phase I and II trials focusing on chronic kidney disease associated with type 2 diabetes [132], a follow-up phase III trial using CDDO-Me was terminated due to undisclosed safety concerns [82,133]. Based on the initially promising therapeutic effect, new compounds related to CDDO-Me are under investigation and were beneficial in an animal setting of brain injury after ischemia reperfusion and in a first-in-human phase I clinical trial, in this case in cancer patients [134,135].

## 7. Conclusions

Taken together, Nrf2 is an important mediator of antioxidant signaling during inflammation. Its function is based mainly on induction of the expression of target genes responsible for detoxifying and anti-oxidant effects, although a role of Nrf2 as a transcriptional repressor is also well established. Dysregulation of Nrf2, leading to hyper- or hypo-activation, frequently due to excessive environmental stress, is often accompanied by the development of chronic inflammatory diseases. New insights into the mechanisms responsible for aberrant Nrf2 function are needed. These include, on the one hand, those caused by overloading of Nrf2-dependent signaling and on the other hand, the processes initiated by mutations in the *Nrf2* gene itself or its regulatory partners such as Keap1, allowing constitutive activation of Nrf2. This will facilitate the more selective use of current therapeutic approaches and point towards potentially new ones.
